# Calcium dobesilate ameliorates hepatorenal injury induced by carbon tetrachloride in mice

**DOI:** 10.22038/IJBMS.2022.61499.13606

**Published:** 2022-02

**Authors:** Elham Hakimizadeh, Ayat Kaeidi, Mohammadreza Rahmani, Mohammad Allahtavakoli, Jalal Hassanshahi

**Affiliations:** 1 Physiology-Pharmacology Research Center, Research Institute of Basic Medical Sciences, Rafsanjan University of Medical Sciences, Rafsanjan, Iran; 2 Department of Physiology and Pharmacology, School of Medicine, Rafsanjan University of Medical Sciences, Rafsanjan, Iran

**Keywords:** Apoptosis, Calcium dobesilate, Carbon tetrachloride, Mice, Oxidative stress

## Abstract

**Objective(s):**

Calcium dobesilate (CaD) has anti-oxidant, anti-inflammatory, and anti-apoptotic effects. In this study, the protective effects of CaD against hepatorenal damage induced by carbon tetrachloride (CCl_4_) in mice were evaluated.

**Materials and Methods:**

Thirty male mice were randomly divided into five groups: Control, CaD 100 mg/kg, CCl_4_, CCl_4_+CaD 50 mg/kg, and CCl_4_+CaD 100 mg/kg. CaD (50 and 100 mg/kg) was administered orally once a day for 4 weeks. The liver and kidney indices (serum creatinine, blood urine nitrogen, alanine aminotransferase, and aspartate aminotransferase levels) were determined. Also, liver and kidney tissue oxidant/anti-oxidant markers (glutathione peroxidase, malondialdehyde, total anti-oxidant capacity, and superoxide dismutase) were measured. Cleaved caspase-3, Bax, cytochrome-c, and Bcl-2 protein levels were measured by immunoblotting method in the liver and kidney tissues. The liver and kidney histopathological changes were evaluated by the Hematoxylin and Eosin (H&E) staining method.

**Results:**

CCl_4_ induced significant oxidative stress and apoptosis in kidney and liver tissues that was concomitant with histopathological abnormalities in these organs in the CCl_4_ group versus the control (*P*<0.05). However, CaD (100 mg/kg) could significantly improve the histopathological change in the liver and kidney tissues of CCl_4_+CaD 100 mg/kg mice versus the CCl_4_ group (*P*<0.05). In addition, CaD (100 mg/kg) attenuated the pro and anti-apoptotic markers in the liver and kidney tissues of CCl_4_+CaD 100 mg/kg mice versus the CCl_4_ group (*P*<0.05).

**Conclusion:**

CaD (100 mg/kg) has a protective effect against hepatorenal injury induced by CCl_4_ at least via its anti-apoptotic and anti-oxidant properties.

## Introduction

Carbon tetrachloride (CCl_4_), an industrial solvent, has been used in animal models to explore renal injury in rats (1, 2). It was indicated that the kidney is not the only objective organ of CCl_4_ toxicity, it causes damage in other organs, such as the liver (3), brain (4, 5), testis (6, 7), lungs (8), and blood (9) by generation of free radicals. During the metabolism of CCl_4_, trichloromethyl (CCL3) free radicals cause lipid peroxidation (10, 11) and trichloromethyl peroxyl radical (CCl_3_O_2_) causes renal injuries (12). Damage by CCl_4_ includes altering the endogenous anti-oxidants in tissues which are manifested by histopathological lesions (13). Furthermore, liver and kidney triglycerides, cholesterol, and free fatty acids increase in the CCl_4_ administration animal (14). Anti-oxidants are essential substances that are capable of guarding the body against free radical-induced oxidative stress damage (15, 16). Calcium 2, 5-dihydroxybenzenesulfonate (calcium dobesilate; CaD) is a vascular protective drug that is used for treatment of diabetic retinopathy and chronic venous insufficiency (17, 18). CaD, as an angioprotective drug, can inhibit platelet activity and reduce blood viscosity and also capillary permeability (19). In addition, recent studies have demonstrated that CaD exerts protective effects against diabetic nephropathy (10). Additionally, CaD has multiple mechanisms of action that include anti-oxidant properties which reduce lipid pre-oxidation caused by free oxygen radicals and anti-inflammatory properties that decrease the release of inflammatory cytokines, such as platelet-activating factor (20-23). Moreover, it has been shown that CaD corrects capillary dysfunctions, decreases free oxygen radicals, increases nitric oxide synthase, and prevents desquamation in endothelium cells (24, 25). Considering the anti-inflammatory and anti-oxidant properties of CaD, we evaluated the protective effect of calcium dobesilate administration against hepatorenal damage induced by CCl_4_ in male mice.

## Materials and Methods

***Animal***

In this experimental study, 30 male BALB/c mice (30±5 g) were acquired from Rafsanjan University of Medical Sciences Animal House, Rafsanjan, Iran. The male BALB/c mice were kept in a room with 12 hr light/dark cycle, a humidity of 45%, and a temperature of 20–23 °C with access to food and water *ad libitum*. The BALB/c mice were maintained in the colony room for one week before starting the experiment. The ethics committee of Rafsanjan University of Medical Sciences approved the study (IR.RUMS.REC.1399.055). Also, this study was done in agreement with the Guidelines for Animal Care and Use (National Institutes of Health Publication No. 85-23) revised in 2010. 

***Drugs***

CCl_4_ (10 mmol/kg) (Sigma Chemical Co., St. Louis, MO, USA) was freshly dissolved in 50% olive oil (1:1) (26) for intraperitoneal (IP) administration. CaD was procured from Sigma-Aldrich Company, Germany. CaD (50 and 100 mg/kg) (27) was freshly prepared each day during the experiment, and it was dissolved in saline solution for oral administration.

***Experimental groups and experimental protocol***

Animals were randomly divided into 5 groups (six mice/group) as follows:

Group 1 (Control): This group received olive oil (as a vehicle of CCl_4_) by IP injection on the 1st day, then an hour later, animals were treated with saline by oral gavage daily for 4 weeks (28).

Group 2 (CaD): This group received olive oil by IP injection on the 1st day, then an hour later, animals were treated with CaD (100 mg/kg) (27) by oral gavage daily for 4 weeks (28).

Group 3 (CCl_4_): This group received CCl_4_ (10 mmol/kg) (26) in 50% olive oil (1:1) by IP injection on the 1st day, then an hour later, animals were treated with saline (oral gavage) daily for 4 weeks (28).

Group 4 (CCl_4_ + CaD 50 mg/kg): This group received CCl_4_ (10 mmol/kg) by IP injection on the 1st day, then an hour later, animals were treated with CaD (50 mg/kg) (27) by oral gavage daily for 4 weeks (28).

Group 5 (CCl_4_ + CaD 100 mg/kg): This group received CCl_4_ (10 mmol/kg) by IP injection on the 1st day, then an hour later, animals were treated with CaD (100 mg/kg) (27) by oral gavage daily for 4 weeks (28).

The mice received a single dose of olive oil or CCl_4_ (10 mmol/kg) on the 1st day. Then an hour later, each animal was given the same volumes of saline or CaD (50 or 100 mg/kg) during the experimental period. 24 hr after the last treatment, mice were anesthetized using diethyl ether. The paw pinch reflex as an indicator of anesthetic depth was checked in all animals just before blood sampling. Serum samples were prepared via blood centrifugation (6000 rpm for 15 min) at room temperature. After blood sampling, mice were sacrificed under deep anesthesia. The liver and kidney tissues were removed immediately and divided into two parts. One part was kept in formalin (10%) for histological evaluations by Hematoxylin and Eosin (H&E) staining. The other parts of liver and kidney tissues were homogenized in ice-cold lysis buffer solution (phosphate buffer solution; PBS) enriched with protease inhibitor cocktail (Sigma, USA). The homogenized samples were centrifuged for 20 min at 6000 rpm (at 4 °C); then the supernatant was harvested and kept at -80 °C until molecular evaluation of oxidative stress and apoptosis biochemical parameters (29-31).

***Biochemical parameters measurement***

Alanine aminotransferase (ALT) and aspartate aminotransferase (AST) serum levels were measured using the relevant kits (Pars Azmoon, Co., Tehran, Iran) by an automatic autoanalyzer (HECTIC, Japan). Serum creatinine (Cr) and blood urine nitrogen (BUN) levels were determined via quantitative commercial kits (Pars Azmoon, Co., Tehran, Iran) (32, 33). 

***Hepatorenal oxidative stress measurement***

To evaluate oxidative stress status in the liver and kidney tissues, the glutathione peroxidase (GPx) and superoxide dismutase (SOD) enzyme activity levels, malondialdehyde (MDA) concentration, and total anti-oxidant capacity (TAC) status were measured via available commercial assay kits (ZellBio, Germany) according to the manufacturer’s instructions using an automatic microplate reader (BioTek, USA) (16, 34). 

***Hepatorenal apoptosis measurement***

The immunoblotting method was done to evaluate cleaved caspase-3, cytochrome-c, Bax, and Bcl-2 proteins expression levels as apoptosis biomarkers in the liver and kidney tissues. In brief, each protein sample was isolated via 12.5% polyacrylamide gel electrophoresis and subsequently transferred to polyvinylidene difluoride (PVDF) membrane by electric current. The PVDF membranes were incubated (overnight at the temperature of 4 °C and pH 7.4) in blocking buffer (20 mM Tris–HCl, 150 mM NaCl, 0.1% Tween 20 with 5% nonfat milk). After 3 times (5 min each at room temperature) washing the membrane with washing buffer (20 mM Tris–HCl, 150 mM NaCl, 0.1% Tween 20), each PVDF membrane was incubated with rabbit polyclonal anti-Bax, anti-caspase-3, anti-cytochrome-c, and anti-Bcl-2 antibodies (1: 1000, Abcam, USA) for 3 hr at room temperature. Subsequently, the blots were washed with washing buffer and incubated with horseradish peroxidase-conjugated goat anti-rabbit secondary antibody (1:5000, Abcam, USA) at room temperature for 1 hr. Then each blot was detected via an enhanced chemiluminescence method. Band densitometry analysis was done using the ImageJ software. Beta (β)-actin immunoblotting (1:5000, Abcam, USA) was applied as a loading control (16, 30).

***Histopathological analysis***

The liver and kidney sections were stained using the routine H&E method (magnification 100×). Each stained slide was observed via a light microscope (Nikon Labophot, Japan) in a blind manner to score kidney and liver tissue damage (KTDS and LTDS) for histopathological analysis. Each kidney section was measured for inflammatory cell infiltration, glomerular atrophy, and tubular necrosis. The kidney tubulointerstitial injury was evaluated semi-quantitatively according to previous studies (16, 30). Also, each liver section was measured for congestion and pyknosis. KTDS and LTDS were graded from 0 to 3 by a pathologist blinded to our study (0-0.5= normal, 1= mild, 2= moderate, and 3= severe) (32, 35, 36).

***Data analysis***

Data were analyzed using the GraphPad Prism software package (ver. 6.01, GraphPad Software, USA). The normality of the results was analyzed using the Kolmogorov-Smirnov test. The results are expressed as mean ± SD. For parametric data, one-way ANOVA followed by Tukey’s test was done to evaluate the significance level between the groups. For non-parametric data, the Kruskal-Wallis test was performed. *P*<0.05 was considered a significant level. 

## Results

***Effect of calcium dobesilate on biochemical parameters***

The results showed that serum Cr, BUN, AST, and ALT levels were increased in the CCl_4_-treated mice compared with the control group (*P*<0.01) (Table 1). 

CaD (50 and 100 mg/kg) did significantly affect BUN as compared with the CCl_4_ administrated group. In addition, CaD (100 mg/kg) reduced the Cr level in the CCl_4_+CaD 100 mg/kg group compared with CCl_4_-treated mice (*P*<0.05). Furthermore, CaD (100 mg/kg) could significantly reduce both AST (*P*<0.05) and ALT (*P*<0.01) levels in the CCl_4_+CaD 100 mg/kg group compared with the CCl_4_ administrated mice (Table 1). However, CaD (50 mg/kg) had no significant effect on AST (P=0.996) and ALT (*P*=0.989) levels in the CCl_4_+CaD 50 mg/kg group versus the CCl_4_ group (Table 1).

***Effect of calcium dobesilate on oxidative stress parameters***

Free radical injury after CCl_4_ administration was evaluated using lipid peroxidation in the kidney and liver tissues, which was assessed by MDA concentration. According to Table 2, CCl_4_ significantly increased the MDA concentration in kidney and liver tissues in mice in comparison with the control group (*P*<0.05). Administration of CaD (100 mg/kg) significantly decreased the MDA concentration in the kidney (*P*<0.05) and liver (*P*<0.01) tissues in the CCl_4_ + CaD 100 mg/kg group versus CCl_4_ mice (Table 2). Moreover, CaD (50 mg/kg) could decrease the MDA concentration significantly in the liver tissue in CCl_4_ + CaD 50 mg/kg group when compared with the CCl_4_-group (*P*<0.05, Table 2).

According to the results, CCl_4_ significantly decreased SOD and GPx activity (as two important anti-oxidant enzymes) in the kidney and liver tissues in the CCl_4_-group compared with the control mice (*P*<0.01, Table 2). As shown in Table 2, administration of CaD (50 and 100 mg/kg) could significantly increase the SOD activity level in the kidney and liver tissues in CCl_4_ + CaD 50 mg/kg and CCl_4_ + CaD 100 mg/kg mice versus CCl_4_ mice (*P*<0.05). Furthermore, CaD (50 and 100 mg/kg) significantly elevated the GPx activity in the liver tissue in CCl_4_ + CaD 50 mg/kg and CCl_4_ + CaD 100 mg/kg mice when compared with CCl_4_ mice (*P*<0.05, Table 2). Also, administration of CaD (100 mg/kg) significantly increased the GPx activity (*P*<0.05) in the kidney of the CaD treated mice compared with the CCl_4_-group. However, no significant difference was seen in the kidney GPx activity level in CCl_4_ + CaD 50 mice versus the CCl_4_ group (Table 2).

According to Table 2, CCl_4_ significantly reduced the TAC level in kidney (*P*<0.01) and liver (*P*<0.05) tissues in mice rather than in controls. Also, CaD (100 mg/kg) could significantly increase the TAC level in kidney and liver tissues in the CCl_4_ + CaD 100 mg/kg group compared with the CCl_4_-group (*P*<0.05, Table 2).

***Effect of calcium dobesilate on kidney and liver apoptosis***

**Figure 1:**
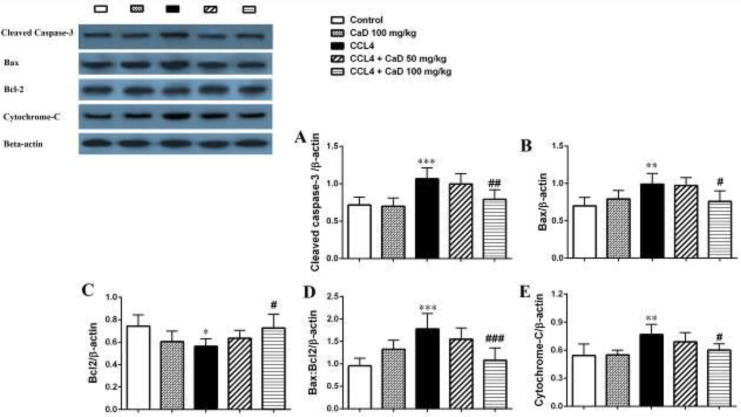
Western blot analysis of the cleaved caspase-3 (A), Bax (B), Bcl-2 (C), Bax/Bcl-2 ratio (D), and cytochrome-c (E) proteins expression in the kidney tissue of mice with CCl_4_ administration. Values are expressed as mean ± SD. n= 6 per group

**Figure 2:**
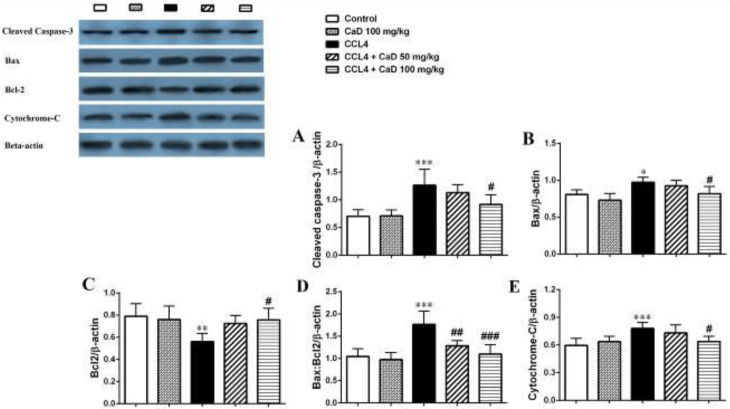
Western blot analysis of the cleaved caspase-3 (A), Bax (B), Bcl-2 (C), Bax/Bcl-2 ratio (D), and cytochrome-c (E) proteins expression in the liver tissue of the mice with CCL4 administration. Values are expressed as mean ± SD. n= 6 per group

Our result also showed that CCl_4_ significantly increased the cleaved caspase-3, Bax, and cytochrome-c proteins expression levels in the kidney and liver tissues in the CCl_4_ administered mice compared with the control group (*P*<0.05, Figures 1 and 2). However, CaD (100 mg/kg) could decrease the cleaved caspase-3, Bax, and cytochrome-c proteins expression levels in the kidney and liver tissues in the CCl_4_ + CaD 100 mg/kg treated group versus the CCl_4_ group (*P*<0.05, Figures 1 and 2).

Moreover, as shown in Figures 1C and 2C, CCl_4_ significantly decreased the Bcl-2 protein expression level in the kidney and liver tissues in the CCl_4_ administered group rather than the control (*P*<0.05). Also, CaD at doses of 100 mg/kg increased the Bcl-2 protein expression level in the kidney and liver tissues in CCl_4_ + CaD 100 mg/kg treated mice compared with CCl_4_ administered mice (*P*<0.05, Figures 1C and 2C).

As shown in Figures 1D and 2D, CaD at the dose of 100 mg/kg significantly decreased the Bax:Bcl-2 ratio in the kidney tissue in the CCl_4_ + CaD 100 mg/kg group compared with CCl_4_ administered mice (*P*<0.001). Also, CaD at doses of 50 and 100 mg/kg significantly decreased the Bax:Bcl-2 ratio in the liver tissue (*P*<0.01 and *P*<0.001, respectively) in CaD-treated groups (CCl_4_ + CaD 50 mg/kg and CCl_4_ + CaD 100 mg/kg) compared with CCl_4_ administered mice.

***Effect of calcium dobesilate on kidney and liver tissue damage***

**Figure 3:**
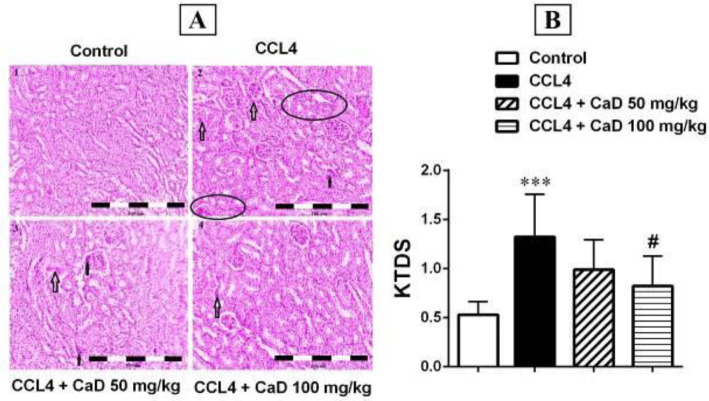
Hematoxylin and Eosin-stained sections (magnification 100×) in the kidney tissue of mice with CCl_4_ administration. Scores 1–3 were considered as leukocyte infiltration, glomerular atrophy, and tubular necrosis in the kidney tissue sections. However, scores 0 to 0.5 were considered normal tissue. B: Kidney tissue damage score (KTDS) in CCl_4_-induced hepatorenal damage mice, four weeks after administration of saline/CaD. Values are expressed as mean ± SD. n= 6 per group. Arrow: glomerular atrophy/tubular necrosis; Circle: leukocyte infiltration

**Figure 4:**
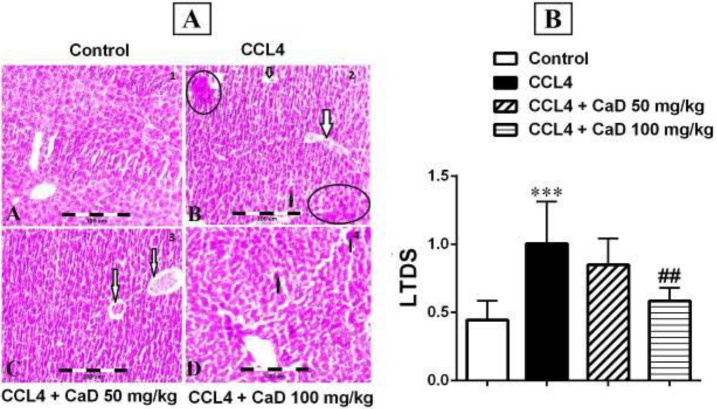
Hematoxylin and Eosin-stained sections (magnification 100×) in the liver tissue of mice with CCl_4_ administration. 1: Scores 1–3 were considered as congestion, fat deposit, and pyknotic cells in the liver tissue sections. However, scores 0 to 0.5 were considered normal tissue. 2: liver tissue damage score (LTDS) in CCl_4_ induced hepatorenal damage mice, four weeks after administration of saline/CaD. Values are expressed as mean ± SD. n= 6 per group. ٭٭٭*P*˂0.001 versus the control group. Arrow: pyknosis/fat deposit; Circle: congestion. ##*P*˂0.01 versus CCl_4_ group

The H&E staining results showed that there was no pathologic condition in the kidney and liver tissues of the control group (Figures 3A and 4A). Furthermore, the histopathologic results showed that CCl_4_ increases kidney and liver damage, so that there were mild and severe kidney and liver damages in CCl_4_ administered mice when compared with the control group (*P*<0.001, Figures 3B and 4B), and CaD (100 mg/kg) could attenuate the kidney and liver tissue damage caused by CCl_4_ (Figures 3A and 4A). Therefore, KTDS and LTDS were significantly decreased in the CCl_4_ + CaD 100 mg/kg treated mice when compared with the CCl_4_ administered group (*P*<0.05 and *P*<0.01, respectively, Figures 3B and 4B). 

**Table 1:** Effect of calcium dobesilate on functional indicators of the kidney and liver in mice with CCl_4_ administration. Values are expressed as mean ± SD n= 6 per group. ٭٭*P*˂0.01 versus the control group. #*P*˂0.05 and ##*P*˂0.01 versus CCL4 group

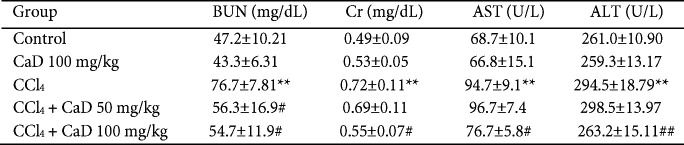

CCl_4_: Carbon tetrachloride, CaD: Calcium dobesilate, BUN: Blood urea nitrogen, Cr: Creatinine, AST: Aspartate aminotransferase, ALT: Alanine transaminase

**Table 2.** Effect of calcium dobesilate on oxidative stress parameters of kidney and liver tissues in mice with CCl_4_ administration. Values are expressed as mean ± SD. n= 6 per group. ٭*P*˂0.05 and ٭٭*P*˂0.01 versus the control group. #*P*˂0.05 and ##*P*˂0.01 versus the CCl_4_ group

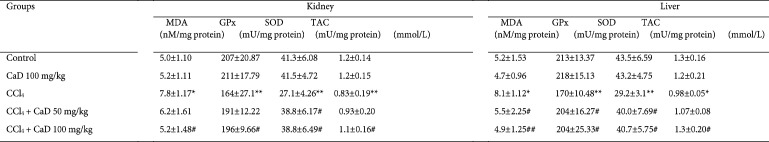

CCl_4_: Carbon tetrachloride, CaD: Calcium dobesilate, MDA: Malondialdehyde, GPx: Glutathione peroxidase, SOD: Superoxide dismutase, TAC: Total antioxidant capacity

*P˂0.05, ٭٭*P*˂0.01, and ٭٭٭*P*˂0.001 versus the control group. #*P*˂0.05, ##*P*˂0.01, and ###*P*˂0.001 versus CCl_4_ group. CCl_4_: Carbon tetrachloride, CaD: Calcium dobesilate

*P˂0.05, ٭٭*P*˂0.01, and ٭٭٭*P*˂0.001 versus the control group. #*P*˂0.05, ##*P*˂0.01, and ###*P*˂0.001 versus CCl_4_ group. CCl_4_: Carbon tetrachloride, CaD: Calcium dobesilate

٭٭٭*P*˂0.001 versus the control group. #*P*˂0.05 versus CCl_4_ group. CCl_4_: Carbon tetrachloride, CaD: Calcium dobesilate

CCl_4_: Carbon tetrachloride, CaD: Calcium dobesilate

## Discussion

In the present study, we examined the protective effects of CaD (50 and 100 mg/kg) against hepatorenal damage induced by CCl_4_ in mice. Our result showed that CCl_4_ increases BUN, Cr, ALT, and AST levels. In line with our study, it has been shown that CCl_4_ treatment causes significant hepatorenal injuries and increases BUN, Cr, ALT, and AST levels (37, 38). Our results also showed that CCl_4_ increases the MDA levels and decreases GPx, SOD activities, and TAC in the liver and kidney tissues. In accordance with our results, it has been reported that CCl_4_ decreases SOD, GPx, and catalase activity in the kidney and also induces oxidative stress in the liver and kidney tissues (37, 38). Moreover, our results showed that administration of CaD at the dose of 100 mg/kg can attenuate the hepatorenal injuries induced by CCl_4_ via reducing BUN, Cr, AST, and ALT levels (Table 1). Also, our results showed that CaD (100 mg/kg) can ameliorate oxidative stress via decreasing MDA concentration and increasing the GPx, SOD activities, and TAC in the liver and kidney tissues of CCl_4_ administrated mice (Table 2). In this regard, previous reports revealed that CaD has anti-oxidant properties (39); Seker *et al*. found that CaD has protective effects against intestinal ischemia-reperfusion injury via increasing the TAC indicator (22). In another study, it was revealed that CaD reduces the retinal injury induced by ischemia/reperfusion by increasing the content of GPx (40). Moreover, it has been shown that CaD is a potent free radical scavenger and reduces the level of MDA (as a marker of lipid peroxidation) (41). Besides, over-generation of MDA could result in pathological changes in the kidneys and liver (30, 42). Our results also showed that CCl_4_ can induce apoptosis via increasing cleaved caspase-3, Bax, and cytochrome-c proteins expression levels, and lowering Bcl-2 protein expression level in the kidney and liver (Figures 1 and 2). However, CaD (100 mg/kg) could attenuate apoptosis in the liver and kidney tissues via at least decreasing the cleaved caspase-3, Bax, and cytochrome-c proteins expression levels and increasing the Bcl-2 protein expression level. In line with our study, Zhang *et al*. reported that CaD has an important role in inhibiting apoptosis factors in the kidney and liver tissues (43). On the other hand, it is specified that oxidative stress triggers apoptosis (44), which results in liver and kidney degeneration (45). In our study, it is possible that CaD (100 mg/kg) also has attenuated the hepatorenal apoptosis induced by CCl_4_ via reducing oxidative stress along with ameliorating the pro- and anti-apoptotic markers. Furthermore, our histopathologic results showed that CCl_4_ increases kidney and liver damage, and CaD (100 mg/kg) could attenuate KTDS and LTDS induced by CCl_4_ (Figures 3 and 4). In the present study, renal tubular necrosis, glomerular atrophy, renal leukocyte infiltration, hepatic congestion, sinusoid dilatation, and pyknosis of hepatocytes, can be regarded as proof of hepatorenal damage in addition to increased abovementioned biochemical parameters in CCl_4_ administered mice. Moreover, the liver and kidney histological results are in accord with the abovementioned changes in the biochemical and oxidative factors. In favor of our results, it has previously been shown that CCl_4_ induces oxidative damage and increases histopathological change in the liver and kidney tissue (46). In this regard, it has been reported that CaD can reduce the renal histopathological change induced by gentamicin and ameliorate nephrotoxicity (47). 

## Conclusion

Our study showed that CaD at the dose of 100 mg/kg can decrease BUN and Cr levels in the CCl_4_ administrated mice. Also, CaD (100 mg/kg) can attenuate the oxidative stress in the kidney and liver tissue of the CCl_4_ administrated mice via its anti-oxidant effect. Moreover, CaD (100 mg/kg) could ameliorate the apoptotic indicators and histopathological changes in the liver and kidney tissues of the CCl_4_ administered mice. Overall, the findings of the present study showed that CaD (100 mg/kg) possibly has a protective effect against CCl_4_ hepatorenal toxicity. However, additional studies must be performed to improve understanding of the mechanisms and degree of this protective effect. 

## Authors’ Contributions

JH Conceived and designed the experiments; EH, AK, and JH Performed the experiments; AK, MR, MA, and JH Analyzed the data; MA, MR, and JH Contributed reagents/materials/analysis tools; and AK, EH, and JH Wrote the article.; All authors agree to be accountable for all aspects of the work, ensuring integrity and accuracy. The authors declare that all data were generated in-house and that no paper mill was used. 

## Conflicts of Interest

The authors declare no conflicts of interest.
